# Promises but Also Perils: Why We Should Not Dismiss Mental Surveillance Risks in Neurotechnology Research

**DOI:** 10.1002/hast.70119

**Published:** 2026-10-28

**Authors:** Andrea Lavazza

**Keywords:** neurotechnology, mental surveillance, mental privacy, psychology, mental integrity, personal identity, research ethics, neuroscience and society

## Abstract

*Speech brain‐computer interfaces may restore communication to people who have lost the ability to speak. In the article “Speech Restoration and Mental Surveillance as Conflicting Ethical Concerns in Neural Speech‐Decoding Research,” Narayan Sankaran and Winston Chiong distinguish systems that decode intended speech from those that might reveal thoughts a person does not wish to communicate. This commentary argues that the surveillance question also extends beyond the device being tested. Research for clinical purposes produces methods, expertise, and infrastructure that could later be adapted for other uses, although present speech interfaces cannot simply be turned into surveillance tools. Mental privacy matters because it protects the space in which people reflect, revise their judgments, and decide what to share. Patients may reasonably accept some privacy risks to communicate, but their choices cannot authorize future uses that affect others. Researchers, funders, and regulators should therefore consider misuse early, involve prospective users in design decisions, and develop safeguards, including international coordination, that preserve access to speech restoration while limiting unwanted neural monitoring*.

## Other Voices

Narayan Sankaran and Winston Chiong, in this issue of the *Hastings Center Report*, examine a difficult tension in neural speech‐decoding research.^1^ Speech brain‐computer interfaces (BCIs) could restore fluent communication to people who have lost the ability to speak. At the same time, progress in decoding speech‐related neural activity raises concerns about mental surveillance. The authors show why the ethical assessment of a device should consider how closely the signals it decodes are tied to a person's intention to communicate. I agree that this distinction is valuable. I want to add a question that remains even when a particular device is designed to decode only intended speech: what responsibilities arise when research conducted for a beneficial purpose makes a new technical capacity available for other uses?

The promise of speech restoration deserves to be taken seriously. For a person who cannot speak, the ability to communicate more fluently may transform relationships, access to care, and participation in social life. Yet the clinical purpose of a research program cannot, by itself, determine how the knowledge and tools it produces will later be used. Sankaran and Chiong acknowledge the historical and commercial reasons for concern about surveillance. My emphasis is on a further aspect of that concern: once a capacity to decode increasingly detailed information from neural activity has been developed, its potential uses may extend beyond those for which it was originally created.

What, then, is the core of the surveillance concern? In developing tools to help people who cannot communicate fluently, we are also learning how to extract increasingly detailed information from brain activity. I am not claiming that current speech BCIs can secretly read anyone's thoughts or that their developers intend to create surveillance devices. Present systems require specific equipment, training, and conditions of use, and their outputs remain fallible. The concern is that the knowledge gained for a good clinical purpose might later be used to pursue a different one.^2^

A simple analogy helps to locate the issue. Suppose a group of people is trapped behind the doors of an underground shelter and a new, exceptionally powerful explosive could free them. We would have strong reasons to develop and use it for the rescue. We would still have reasons to control what happens to that capacity afterward. Speech decoding is unlike an explosive in many respects. A compelling reason to develop a tool does not settle the terms of its later availability.

The benefits and risks of this research are not simultaneous. The prospective benefit to a person who cannot speak is concrete and urgent; a possible surveillance use is less certain and may lie years away. This difference understandably gives restoration greater weight in present decisions. Yet uncertainty about a future use should not be confused with an absence of responsibility for it. Research decisions help determine which capacities will exist, how widely the relevant expertise will be distributed, and which applications will become technically and economically attractive. These decisions are easier to shape while a technology is still being developed than after devices, trained models, and established practices have spread.^3^ Taking surveillance concerns seriously means asking how to preserve the clinical benefit while keeping future choices open to public scrutiny and control.

Why does this possibility matter so much? The mind may be the last frontier of our privacy, a sanctuary of our individuality. Our movements, communications, and relationships already leave traces that others can collect and analyze. But we have generally been able to keep a thought to ourselves, even when we could not control what happened around us. A person subjected to pressure or coercion may still preserve private convictions, memories, and plans. This inner space allows us to reflect, doubt, and resist.^4^

Mental privacy protects more than information. We need room to consider competing values, revise an initial judgment, and decide which commitments to endorse. This process often includes impulses or possibilities that we eventually reject. If tentative thoughts could be exposed, they might be mistaken for settled convictions. Knowing that one's reflections were being observed could itself encourage conformity and change the course of deliberation. In this sense, protecting mental privacy also helps protect moral autonomy.

The same point applies to personal identity. We develop an understanding of ourselves through memories, plans, and narratives that change over time and through our relationships with others. Sharing parts of this inner life is often essential to those relationships. But people also need some control over when and how they share them. Access to a thought before a person has decided whether to express it may affect how others see that person and how the person comes to understand themselves.

The accuracy of a decoder cannot, by itself, settle concerns about mental surveillance. Accuracy matters, and exaggerated claims about “mind reading” should be corrected. But even an imperfect inference about what someone thinks can be intrusive, especially when treated as a reliable account of that person. The concern also extends to the possibility that capacities developed in one setting may be adapted to others as devices become easier to use. Whether and how this will occur are empirical questions, not settled predictions.

It may be objected that present devices generally require the user's participation and operate in controlled settings. That is true. But these conditions might change. Direct neural communication might one day be attractive in military or emergency operations, where speaking is difficult or unsafe. Employers might require workers to use neural monitoring devices to track fatigue and attention to the task in the name of workplace safety. These applications differ from decoding private thoughts, and none should be treated as an inevitable development. They do show how neural monitoring could gradually become acceptable in settings where declining it carries a professional cost. In such circumstances, how voluntary would participation really be?

The effects of monitoring may also change as it becomes familiar. José Muñoz calls for research into whether people who routinely use neurotechnologies learn to regulate their thoughts and reactions in response to what a device might reveal.^5^ We do not yet know whether this happens, but the question matters. If workers came to regard the monitoring of neural activity as an ordinary condition of their job, privacy could be weakened even without anyone using a device to decode a specific private thought.

There is a further complication: people may reasonably differ in how much protection they want. Someone who has lost the ability to speak might prefer a system that decodes inner speech if it allows faster, less tiring communication, even though it may create a greater risk of disclosing thoughts they did not intend to share. Respect for autonomy requires considering that preference. More generally, some people may willingly accept substantial access to their neural activity for enhancement or closer integration with a digital device. A uniform prohibition on such uses would require justification.^6^

Yet agreeing to one form of access does not mean giving up control over all others. A person may consent to a BCI decoding inner speech while the device is switched on, but object to continuous recording, long‐term storage, or the use of those data to train systems for other purposes. Nor can consent always resolve the broader risks: some effects may be difficult to anticipate or reverse, and some uses may affect other people's rights. In speech restoration, the practical question is how to offer users meaningful choices about the device's operation while maintaining limits on uses they have not authorized. Patients should be able to accept a degree of privacy risk in order to communicate without having that decision treated as a general waiver of mental privacy.

There is also a limit to what any individual choice can authorize. A prospective user may decide that the benefits of a particular speech BCI justify its privacy risks for them. That decision deserves respect, but it cannot settle the risks that arise if the methods developed for that device are later adapted for use on others. Equally, those wider risks should not be placed entirely on the shoulders of people seeking to regain communication. Assessing the risks is a responsibility for researchers, funders, regulators, and the wider public.

I have argued for a right to mental integrity that would protect people both from unwanted access to their mental states and from direct interference with their mental processes.^7^ The legal boundaries of such a right still need to be defined. Nevertheless, it offers a clear starting point: protection should be considered while neurotechnologies are designed, rather than added only after a device has been developed. Devices could be designed to signal and, where possible, prevent unauthorized collection or transmission of brain data.^8^ Users should have effective control over when decoding begins and what information leaves the device. A recent experimental speech BCI, for example, used an activation phrase imagined by the user before decoding inner speech.^9^ Such a feature does not resolve every privacy problem, but it shows that protection can be treated as a design requirement.

Protecting the neural data of current users is indispensable, but it addresses only part of the concern. A speech BCI developed and trained for a particular person does not thereby become a device that can monitor anyone else. Its performance depends on the signals recorded, the person's participation, the data used for training, and the setting in which it operates. Moving from clinical communication to surveillance would require further research and substantial adaptation. These obstacles should be stated clearly, rather than treated as minor details.

Still, research produces methods, expertise, and infrastructure that can be developed further. Investigators learn which neural signals contain useful information, how to record them, and how to train systems to interpret them. Future devices may use this knowledge under different conditions and for different purposes.^10^ Governance therefore needs to consider the protection of data collected from present users and the possible development of the methods created through the research. The relevant questions are how a device's output and underlying data may be used and under what conditions a decoding method should be tested or deployed beyond its original clinical purpose. Asking these questions early does not require predicting that surveillance will occur. It allows us to assess a foreseeable possibility before a new application has become established.

Institutional decisions also matter. Researchers, funders, and regulators could assess whether a proposed system can decode information beyond what its user intends to communicate and whether that capacity is necessary for its clinical purpose. Where a device has substantial potential for uses outside that purpose, stronger conditions for access, testing, or deployment may be warranted. These conditions must be specified carefully so that protecting privacy does not needlessly obstruct research or restrict access for people who need communication aids. Patients and prospective users should participate in deciding which safeguards work in practice.^11^

No single technical or regulatory measure will eliminate the possibility of misuse, especially by actors unwilling to respect such conditions. International coordination is needed, particularly as advances in AI may improve neural decoding and make methods developed in one country easier to adapt elsewhere. Shared standards for the design, testing, and transfer of systems with substantial decoding capabilities could make harmful uses harder to pursue and easier to identify. They would not bind every actor or remove the need for national oversight. They would, however, help prevent protections established in one jurisdiction from being bypassed simply by moving development or deployment to another jurisdiction.

Sankaran and Chiong help us distinguish neural signals more closely linked to a person's intention to speak from signals that may also reveal information the person wishes to keep private. This is an important step. But the question cannot end with the properties of the device now being tested. We must also consider what the research makes possible for others to build later, and who will decide how those possibilities are used. Restoring speech is a compelling goal. Precisely because it is so compelling, we should pursue it in ways that also protect the private mental space on which freedom of thought depends.

## Acknowledgment

This paper is part of a series, Neuroscience and Society, whose development by The Hastings Center for Bioethics is funded by the Dana Foundation.

## AI‐Use Disclosure

Before submission, the author used GPT 5.6 Sol for assistance with translation and editorial revision. The author reviewed and takes responsibility for the final text.
